# Incidence and predictors of cardiovascular outcomes after acute coronary syndrome in a population-based cohort study

**DOI:** 10.1038/s41598-023-30597-w

**Published:** 2023-03-01

**Authors:** Anders Ulvenstam, Anna Graipe, Anna-Lotta Irewall, Lars Söderström, Thomas Mooe

**Affiliations:** Department of Public Health and Clinical Medicine, Umeå University, Umeå, Sweden

**Keywords:** Acute coronary syndromes, Vascular diseases, Cerebrovascular disorders, Stroke

## Abstract

There is limited data on long-term outcomes after hospitalization for ACS. We aimed to estimate the rate of recurrent cardiovascular events in the long-term, in a population-based, unselected cohort of ACS patients. We included 1379 patients with ACS hospitalized at Östersund hospital 2010–2014 and followed them from the day after discharge to 31 December 2017. The primary endpoint was the unadjusted rate of the composite of CV death, AMI and ischemic stroke. Risk factors for the primary endpoint were assessed in a multivariable Cox proportional hazards regression model. During a median follow-up of 4.7 years, the unadjusted rate of the primary endpoint was 10.3% at 1 year and 28.6% at the end of follow-up. Predictors of increased risk for subsequent events were congestive heart failure, diabetes mellitus, angina pectoris, prior revascularization with PCI or CABG and treatment with diuretics at discharge. Lipid-lowering therapy at discharge and revascularization with PCI or CABG were associated with a lower risk of recurrent events. The risk of recurrent cardiovascular was high at 1 year and continued to be so during the following almost 3 years of median follow-up. Established predictors of cardiovascular risk were confirmed.

## Introduction

Incidence and mortality of cardiovascular (CV) disease have decreased during recent decades^1^. Improved acute treatment as well as effective secondary prevention have contributed to this positive development^2^. However, ischemic heart disease and stroke still constitute the two leading causes of death, both in Sweden and worldwide^3,4^. There is limited data on long-term outcome after an acute coronary syndrome (ACS) in recent years. Most of our current knowledge on long-term risk after ACS is based on clinical trials, generally not mirroring the general population and with relatively short follow-up periods. Register based studies have study samples more representative of the general population, but they have an inherent weakness in the lack of adjudication of outcome events, which can lead to both under- and overestimation of endpoints, and thereby false risk estimates. Both clinical trials and register-based studies point at a substantial risk elevation for recurrent events in both the short and long terms after ACS^5,6^. Moreover, improved survival after ACS in combination with a growing and ageing population will lead to an increased burden of coronary heart disease^7^. These findings highlight the importance of optimized care, both in the acute phase and long term regarding secondary prevention of recurrent events. A reliable estimate of long-term risk in a contemporary post-ACS population is therefore important in order to be able to direct adequate secondary preventive measures. The aim of this study was to assess the risk for recurrent CV events in an unselected cohort of patients hospitalized for ACS, i.e. acute myocardial infarction (AMI) and unstable angina (UA), with the possibility of long-term follow-up.

## Methods

### Study population and data collection

**Figure 1:**
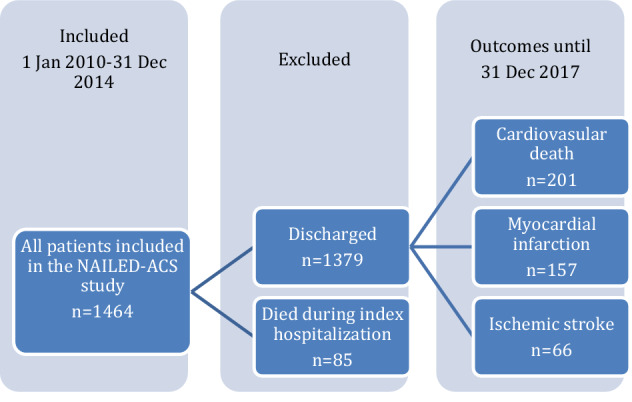
Study flow chart.

We included all patients registered for potential inclusion in the NAILED-ACS Risk Factor Trial, which is a randomized, controlled trial conducted at Östersund Hospital, designed to investigate if intervention by a telephone-based, nurse-led secondary prevention programme could improve CV outcomes after ACS, compared with usual care. Traditional modifiable risk factors were monitored during follow-up for randomized patients in both groups. Patients registered, but not randomized were not monitored during follow-up. The study protocol has been published elsewhere^8^. In summary, the trial included all patients hospitalized for ACS at Östersund Hospital between 1 January 2010 and 31 December 2014. Suspected ACS patients were identified through review of patient records and included if they fulfilled the criteria for UA, defined as suspected ischemic chest pain and electrocardiogram changes suggestive of myocardial ischemia, such as ST-segment depression or T-wave alteration, or type 1 AMI according to the third universal definition of MI. A study flow chart is presented in Fig. 1.

Östersund Hospital is the only hospital in the county of Jämtland/Härjedalen, with a catchment area of about 126.000 people. During the inclusion period, the primary reperfusion treatment for ST-segment myocardial infarction was thrombolysis. In May 2015, Östersund hospital established a 24/7 primary percutaneous coronary intervention (PPCI) network in the county of Jämtland/Härjedalen, which resulted in a switch in reperfusion treatment for STEMI from thrombolysis to PPCI in the vast majority of cases.

Endpoints were identified and adjudicated by reviewing all study subjects discharge records from subsequent hospitalizations at the department of internal medicine and cardiology at Östersund Hospital. Cause of death was adjudicated by reviewing death certificates as well as patient records and classified according to prespecified criteria. To identify endpoints that took place in other departments, relevant International Statistical Classification of Diseases and Related Health Problems (ICD-10) diagnoses were extracted from the national patient register (NPR), which contains main and secondary diagnoses for all hospitalizations in Sweden. These were also adjudicated by review of patient records. The adjudication of events was performed by four experienced physicians (2 senior consultants and 2 senior residents) who were part of the study team. Each physician worked independently according to a standardized workflow algorithm and endpoints were adjudicated strictly according to a prespecified endpoint definition. Consecutive meetings were held where complicated cases were discussed and consensus reached.

### Endpoints

The primary endpoint was major adverse cardiovascular events (MACE) defined as the composite of CV death, type 1 AMI and ischemic stroke, whichever happened first. The follow-up period was from the day after discharge from the index event hospitalization until 31 Dec 2017. All diagnoses were classified according to ICD-10 codes. Death was classified as CV, non-CV or unknown cause of death. CV death was defined as death from CV causes within 30 days after an AMI, sudden cardiac death (SCD), congestive heart failure, stroke, CV procedures, CV bleeding and other CV causes such as pulmonary embolism and peripheral artery disease^9^. AMI was defined according to the third universal definition of myocardial infarction. Only type 1 AMIs were included, where type 1 AMI is defined as an AMI classically caused by plaque rupture, erosion, ulceration or dissection followed by thrombosis at the lesion or distal embolization causing myocardial ischemia and necrosis^10^. Ischemic stroke was defined as an acute episode of focal or global cerebral, spinal or retinal dysfunction caused by infarction of central nervous system (CNS) tissue.

### Statistical analysis

Baseline data for the cohort are presented as means for continuous variables and categorical variables as frequencies. Age is presented as a median and interquartile range (IQR). Baseline characteristics were tested for differences between subjects reaching the primary endpoint or not, with the independent samples T-test or Mann–Whitney U-test as appropriate for continuous variables and the Pearson Chi-square-test for categorical variables.

Kaplan–Meier analysis was used to estimate the cumulative probability of the primary endpoint. Cumulative Kaplan–Meier (KM) incidence was also estimated according to age stratification < 60, 60–69, 70–79 and ≥ 80 years. The individual components of the primary end-point were analyzed separately.

Risk factors for the combined endpoint were analyzed in a Cox proportional hazards model. The proportionality of hazards was verified with Schoenfeld residuals. Variables that previously have been shown to impact risk or otherwise deemed important, were analyzed in univariable analysis and included in the multivariable model if the p-value reached a significance level of < 0.1. Results are presented as hazard ratios (HR) and 95% confidence intervals (CI). Since there was a high overall death rate, and non-CV death might occur before the primary endpoint, a competing risk analysis according to Fine and Gray was performed. Statistical analysis was performed i SPSS (version 25.0; IBM Corp, Armonk, NY) and SAS software (version 9.4; SAS Institute Inc, Cary, NC, USA).

### Ethics

This study was approved by the Regional Ethical Committee in Umeå on October 28, 2009 (Dnr: 09-142M), with supplements on June 10, 2013 (Dnr: 2013-204-32M) and January 13, 2015 (Dnr: 2014-416-32M). This study was conducted according to relevant guidelines and regulations. All study participants had to sign an informed consent form prior to randomization.

## Results

### Baseline characteristics

1379 ACS-patients were included (63.9% men and 36.1% women) and followed from the day after discharge from the index event hospitalization for ACS until 31 Dec 2017. Baseline characteristics are presented in Table 1. The median age was 71 years, with a considerably higher median age in the female population. At index hospitalization, 18.9% were current smokers and 40.8% were previous smokers. 28.4% reported a family history of CV disease defined as a first degree relative with CV disease before the age of 55, or a second degree relative with CV disease before the age of 65. Index ACS-type were NSTEMI in 62.9%, STEMI in 29.5% and UA in 7.6% of cases. 58.5% had hypertension, 22.1% diabetes and 8.6% atrial fibrillation. 21.4% had previously had an MI and 5.9% an ischemic stroke. 53.8% were revascularized during index hospitalization (43.8% PCI and 10% CABG). 60.1% of patients underwent echocardiographic examination during index hospitalization. Of these, 55.4% had a normal ejection fraction (EF), 24.5% were mildly reduced, 14.8% moderately reduced and 5.3% severely reduced. Generally, patients were pharmacologically well treated at discharge. 92.7% had aspirin, 78.5% a P2Y12-inhibitor, 8.2% oral anticoagulation (OAC), 88% betablocker, 87% lipid lowering therapy and 77.3% RAAS-blockade. Medication on admission and at discharge is presented in Tables 2 and 3 respectively.

**Table 1:** Baseline patient characteristics among 1379 patients discharged after hospitalization for ACS.

Variable	Percentage
Age, median (IQR)	71 (± 8.5)
Age, median (IQR) males	68 (± 8)
Age, median (IQR) females	76 (± 8.3)
< 60 years, n (%)	237 (17.2)
60–69 years, n (%)	396 (28.7)
70–79 years, n (%)	377 (27.3)
> 80 years, n (%)	369 (26.8)
Women, %	36.1
Current smoking, %	18.9
Previous smoking, %	40.8
Family history of CV disease, %	28.4
ACS type	
UA, %	8.0
NSTEMI, %	63.6
STEMI, %	28.4
Comorbidities	
Hypertension, %	58.5
Diabetes Mellitus, %	22.1
Atrial fibrillation, %	8.6
Medical history	
Prior AMI, %	21.4
Prior CHF, %	5.9
Prior IS, %	5.9
Prior hemorragic stroke, %	0.6
Prior TIA, %	2.3
Prior peripheral artery disease, %	2.6
Prior PCI, %	8.5
Prior CABG, %	8.7
Prior renal failure %	29
Ejection fraction on echocardiography during index hospitalization (available in 60.1% of patients)	
Normal (> 50%), %	55.5
Mildly reduced (40–49%), %	24.5
Moderately reduced (30–39%), %	14.8
Severely reduced (< 30%), %	5.3
Revascularization during index hospitalization	
Thrombolysis (STEMI), %	67.1
PCI, % (including Rescue PCI)	43.8
CABG, %	10.0

*CV* cardiovascular, *UA* unstable angina, *NSTEMI* non-ST-segment elevation myocardial infarction, *STEMI* ST-segment elevation myocardial infarction, *CHF* congestive heart failure, *TIA* transitory ischemic attack, *PCI* percutaneous coronary intervention, *CABG* coronary artery bypass graft.

**Table 2:** Medication on admission among 1379 patients discharged after hospitalization for AC.

Drug	Percentage
Aspirin (%)	36.3
P2Y12-inhibitor (%)	1.7
Clopidogrel (%)	1.5
Ticagrelor (%)	0.1
Other (%)	0.1
OAC (%)	4.6
Betablocker (%)	38.8
Statin (%)	29.9
ACE-inhibitor (%)	23.4
AT2-antagonist (%)	15.2

*OAC* oral anticoagulation, *ACE* angiotensin converting enzyme, *AT2* angiotensin 2.

**Table 3:** Medication at discharge among 1379 patients discharged after hospitalization for ACS.

Drug	Percentage
Aspirin (%)	92.7
P2Y12-receptor inhibitor (%)	78.5
Clopidogrel (%)	50.5
Ticagrelor (%)	21.9
Other (%)	6.1
OAC (%)	8.2
Betablocker (%)	88.0
Statin (%)	87.0
ACE-inhibitor (%)	60.0
AT2-receptor antagonist (%)	17.3

*OAC* oral anticoagulation, *AT2* angiotensin 2.

### Primary endpoint

**Figure 2:**
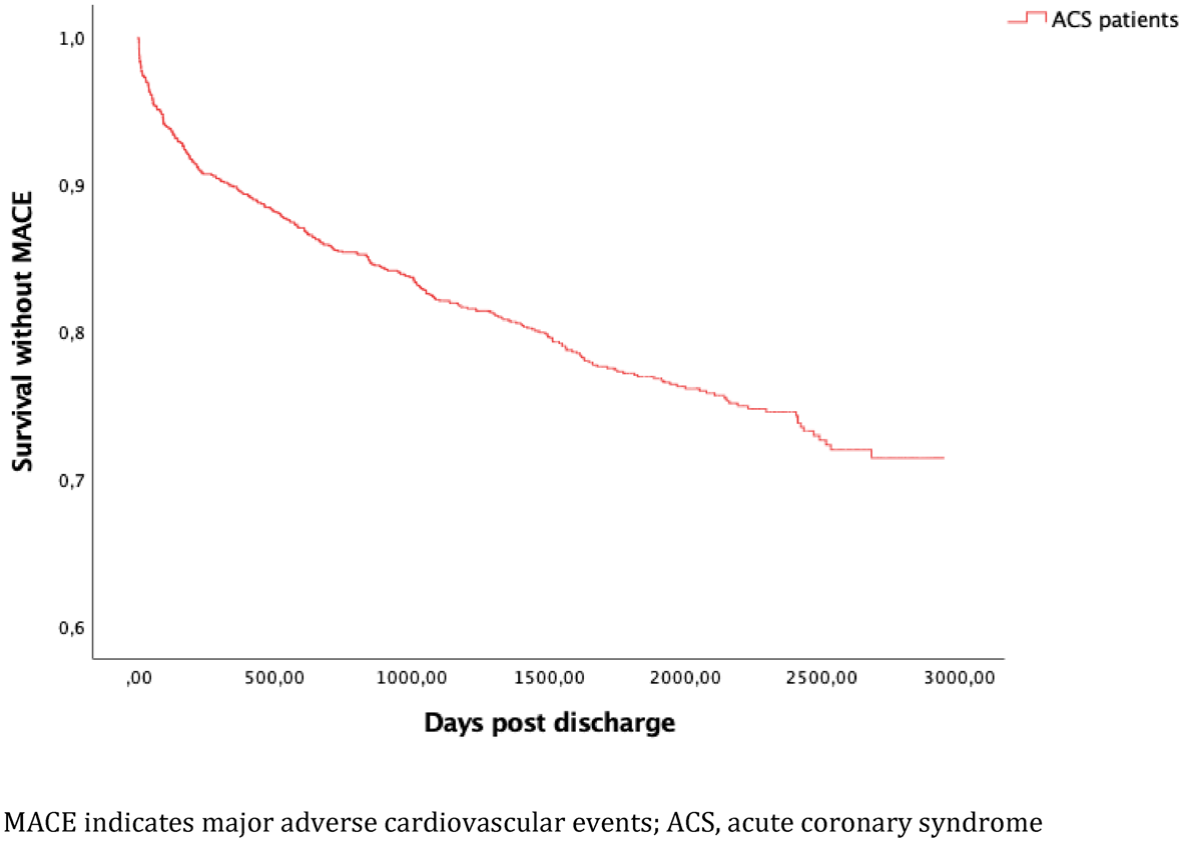
Cumulative Kaplan–Meier estimate of survival without MACE among 1379 patients discharged after hospitalization for ACS between 1 Jan 2010 and 31 Dec 2014.

**Figure 3:**
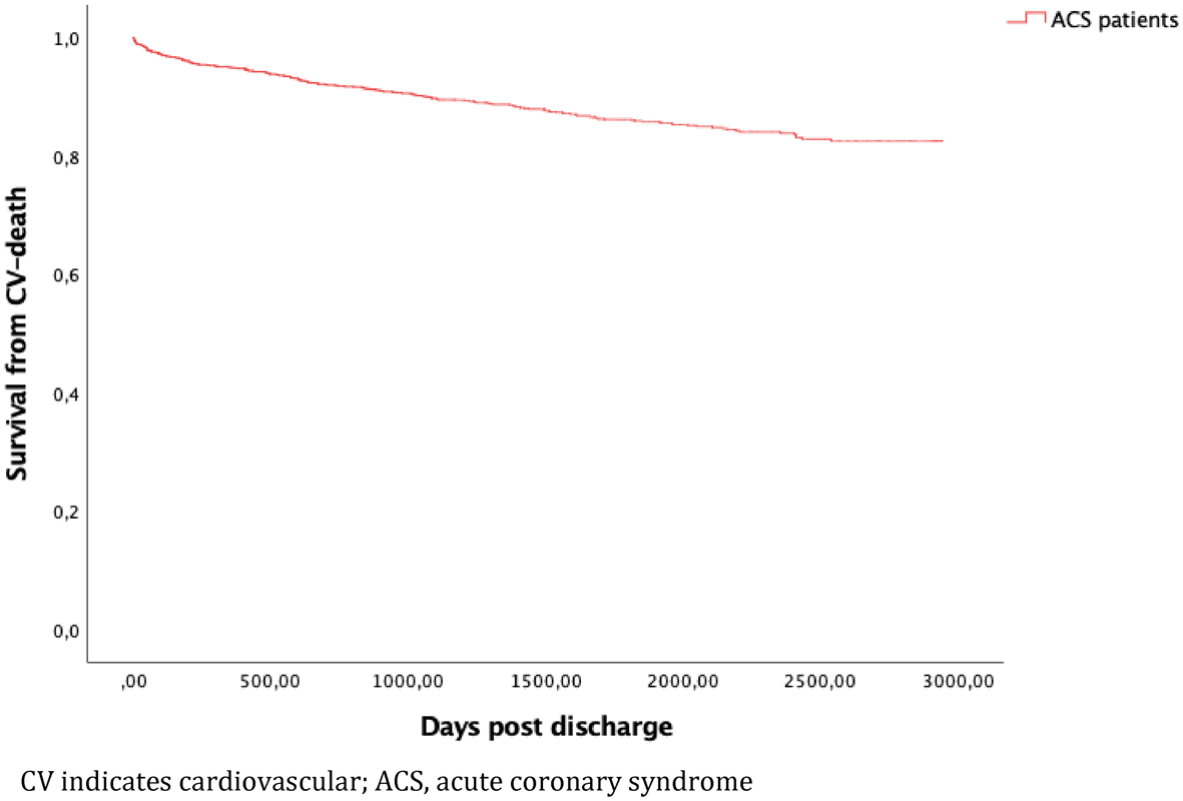
Cumulative Kaplan–Meier estimate of CV-death free survival among 1379 patients discharged after hospitalization for ACS between 1 Jan 2010 and 31 Dec 2014.

**Figure 4:**
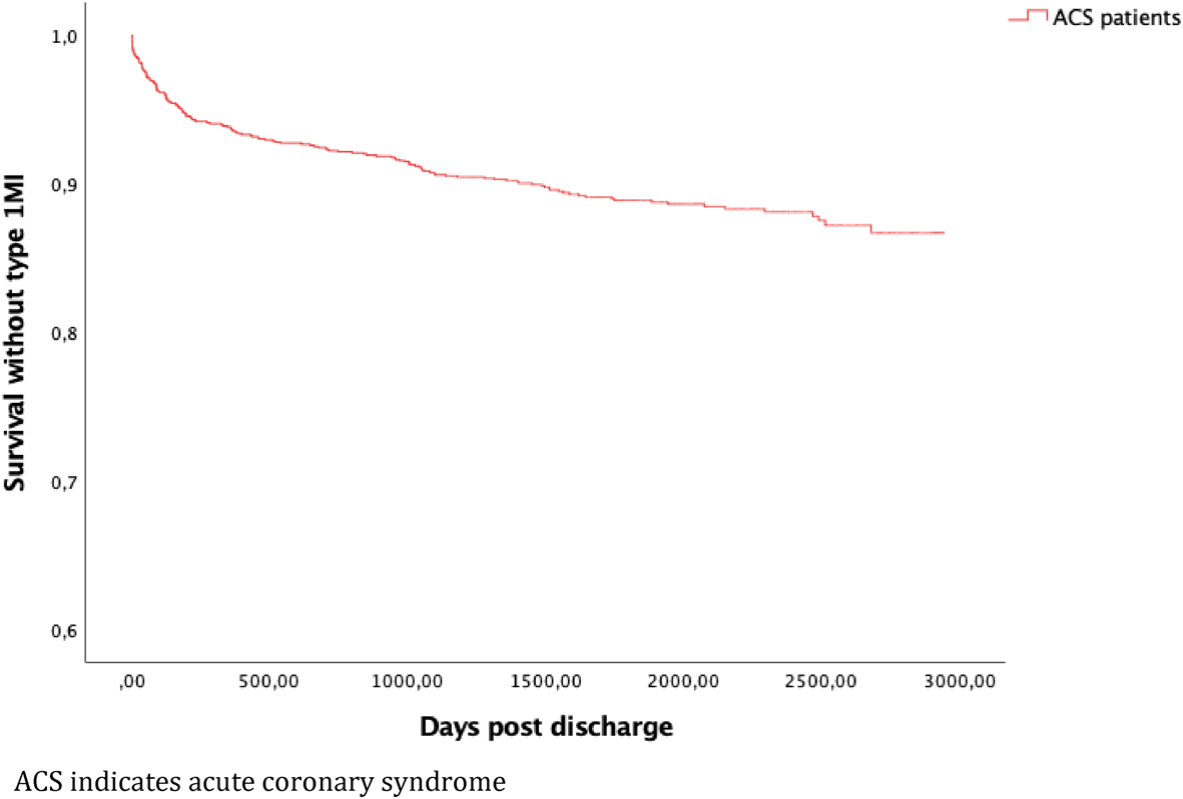
Cumulative Kaplan–Meier estimate of survival without type 1 myocardial infarction among 1379 patients discharged after hospitalization for ACS between 1 Jan 2010 and 31 Dec 2014.

**Figure 5:**
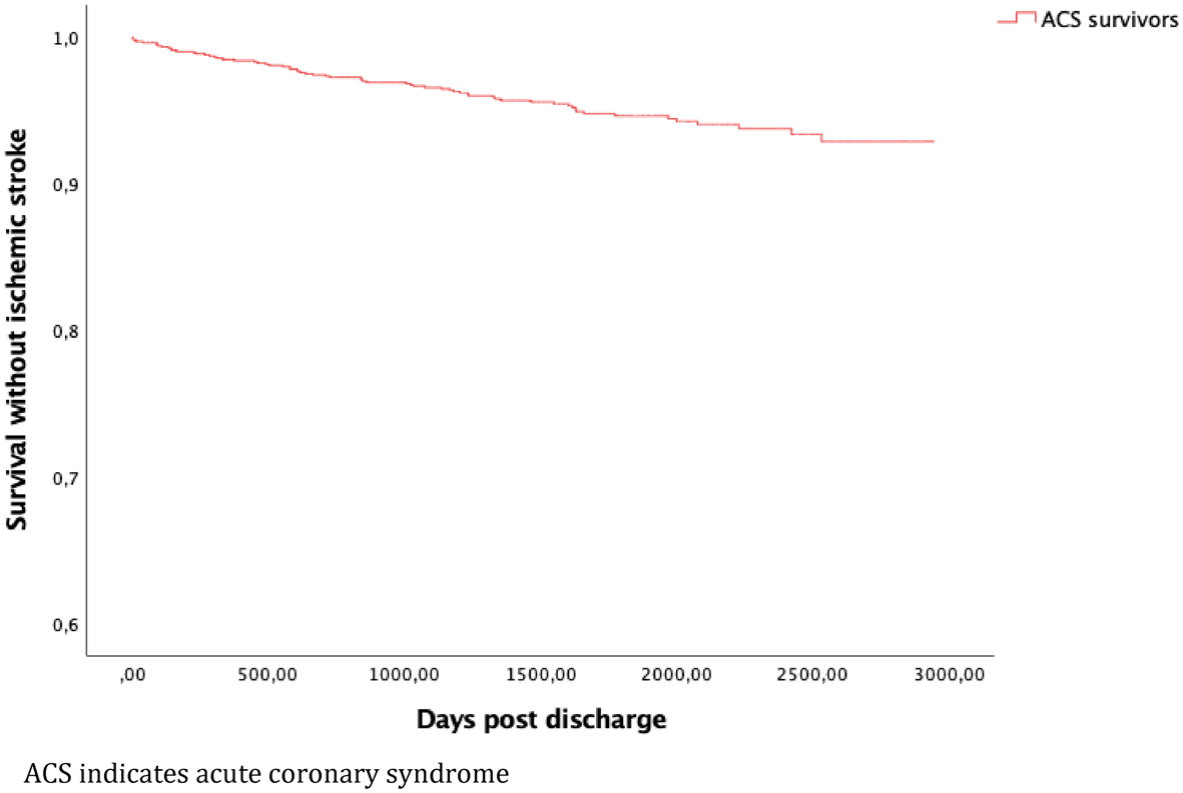
Cumulative Kaplan–Meier estimate of survival without ischemic stroke among 1379 patients discharged after hospitalization for ACS between 1 Jan 2010 and 31 Dec 2014.

In total, there were 327 events meeting the criteria for the primary endpoint during the entire study period, with a median follow-up time of 4.7 years, K–M cumulative incidence 28.6% (95% confidence interval (CI) 26.2–31.0) (Fig. 2). At 1 year, K-M cumulative incidence was 10.3% (95% CI 8.7–12.1). Outcomes including all-cause death at 1 and 4.7 years is presented in Table 4. The K–M incidence of the individual components of the primary endpoint are presented in Figs. 3, 4 and 5 respectively.

**Table 4:** Outcomes at 1 and 4.7 years among 1379 ACS patients.

Event/follow-up time	K-M incidence at 1 year, % (n)	95% confidence interval	K–M incidence at 4.7 years, % (n)	95% confidence interval
MACE	10.3 (142)	8.7–12.1	28.6 (327)	26.2–31.0
CV-death	5.1 (70)	4.0–6.4	17.5 (201)	15.5–19.6
AMI	6.3 (87)	5.1–7.7	13.3 (157)	11.5–15.2
IS	1.5 (20)	0.1–2.3	7.1 (66)	5.8–8.6
All-cause death	8.4 (115)	7.0–10.0	39.2 (404)	36.6–41.8

*MACE* major adverse cardiovascular event, *CV* cardiovascular, *AMI* acute myocardial infarction, *IS* ischemic stroke.

### Risk according to age

**Figure 6:**
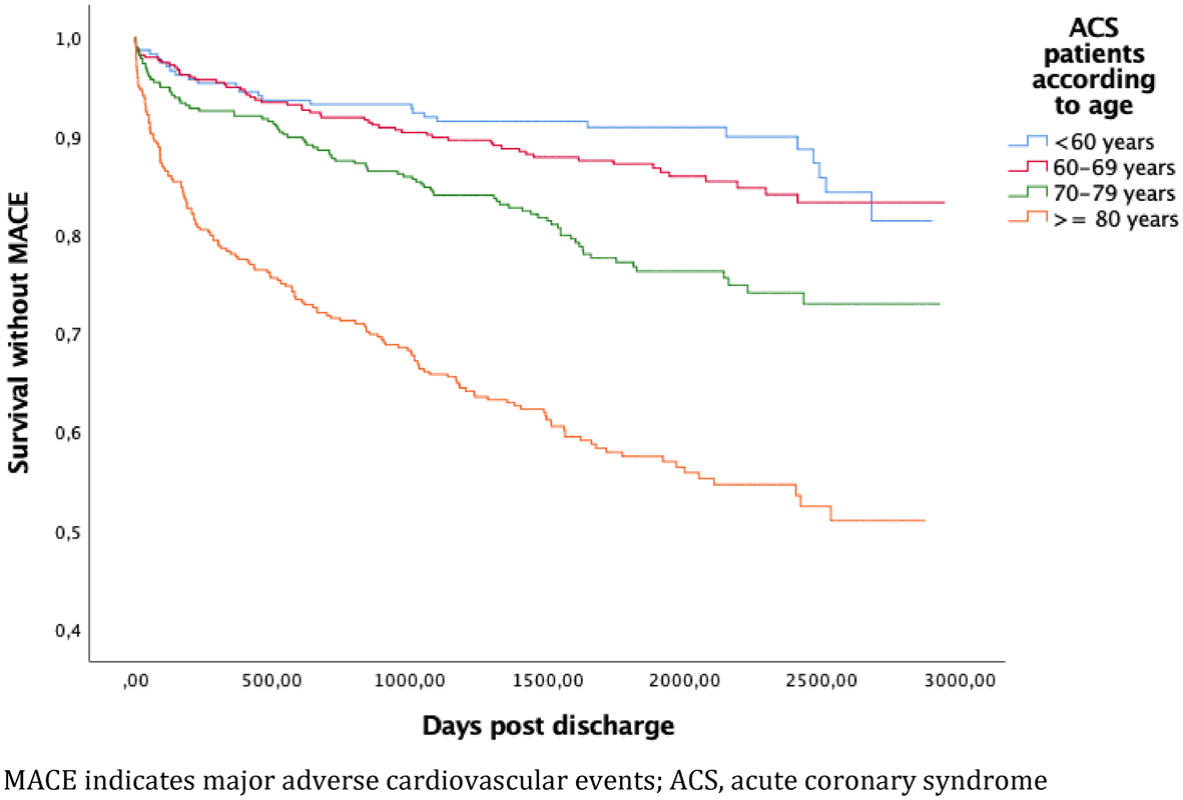
Cumulative Kaplan–Meier estimate of survival without MACE according to age groups among 1379 patients discharged after hospitalization for ACS between 1 Jan 2010 and 31 Dec 2014.

KM estimates for the primary end-point according to age stratification were analyzed at 3 years, due to few events in two youngest age strata towards the end of the studied time period. At 3 years, there were 19 events, KM estimate 8.1% (95% CI 5.1–12.5) among patients < 60 years (n = 237), 40 events, KM estimate 10.2% (95% CI 7.4–15.7) among patients 60–69 years (n = 396), 60 events, KM estimate 16% (95% CI 12.5–20.1) among patients 70–79 years (n = 377) and 126 events, KM estimate 34.2% (95% CI 29.4–39.3) among patients ≥ 80 years (n = 396), see Fig. 6.

### Predictors of the primary endpoint

Baseline characteristics according to the occurrence of the primary endpoint are displayed in Table 5. Patients suffering a primary endpoint event were significantly older and more likely female. Index ACS-type was predominantly NSTEMI among patients reaching an endpoint, who also had significantly more comorbidities such as hypertension, diabetes mellitus and atrial fibrillation. There was also a clear overrepresentation of prior AMI, congestive heart failure (CHF), ischemic stroke and peripheral artery disease among patients experiencing an event. Event-free patients were also revascularized to a far greater extent.

**Table 5:** Patient Characteristics among 1379 patients discharged after hospitalization for ACS according to the occurrence of MACE during the entire follow-up period.

MACE status	No MACE	MACE	p value
Age, median (IQR)	69(± 7.5)	77 (± 8.5)	< 0.001
Women, %	33.4	42.5	0.003
Current smoking, %	20.6	14.8	0.002
Previous smoking, %	43.0	38.2	0.002
ACS type			
UA, %	7.9	8.3	0.831
NSTEMI, %	62.1	68.5	0.035
STEMI, %	30.0	23.2	0.017
Comorbidities			
Hypertension, %	54.3	69.4	< 0.001
Diabetes Mellitus, %	17.8	34.3	< 0.001
Atrial fibrillation, %	14.3	25.1	< 0.001
Medical history			< 0.001
Prior AMI, %	16.1	37.3	< 0.001
Prior CHF, %	2.7	12.8	< 0.001
Prior IS, %	3.5	11.9	< 0.001
Prior hemorragic stroke, %	0.4	0.9	0.233
Prior TIA, %	1.8	3.4	0.092
Prior peripheral artery disease, %	1.8	4.9	0.002
Prior PCI, %	7.1	13.8	< 0.001
Prior CABG, %	5.1	20.2	< 0.001
Prior renal failure, %	22.2	44.6	< 0.001
Treatment in-hospital			
Thrombolysis (STEMI), %	68.7	60.5	0.175
PCI, %	52.4	25.8	< 0.001
CABG, %	11.6	5.5	0.001

*NSTEMI* non ST-segment elevation myocardial infarction, *STEMI* ST-segment myocardial infarction.

Variables tested in a univariable cox regression model are presented in Table 6. In a multivariable model, age HR 1.03 (95% CI 1.02–1.05), prior CHF HR 1.56 (95% CI 1.11–2.21), prior diabetes mellitus HR 1.74 (95% CI 1.36–2.22), prior angina HR 1.41 (95% CI 1.08–1.85), prior PCI HR 1.41 (95% CI 1.00–2.00), prior CABG HR 1.82 (95% CI 1.36–2.49) and treatment with diuretics at discharge HR 1.32 (95% CI 1.04–1.68) were independently associated with an increased risk of the primary endpoint during the study period. Revascularization during index event hospitalization with PCI HR 0.58 (95% CI 0.44–0.77) and CABG HR 0.51 (95% CI 0.31–0.85) as well as lipid lowering therapy at discharge HR 0.69 (95% CI 0.51–0.94) were associated with a reduced risk for the occurrence of the primary endpoint. The result of the multivariable cox regression model is presented in Table 7.

**Table 6:** Risk factors associated with CV-death, MI and IS in 1379 patients discharged after hospitalization for ACS in a univariable analysis.

Variable	HR	p-value
Age	1.058	0.000
Female gender	1.412	0.002
Current or previous smoking smokebin	0.672	0.000
Unstable angina	1.073	0.727
NSTEMI	1.279	0.039
STEMI	0.731	0.017
Family history of CV disease	0.752	0.041
Hypertension	1.786	0.000
Diabetes mellitus	2.111	0.000
Atrial fibrillation	1.856	0.000
Previous myocardial infarction	2.554	0.000
Previous ischemic stroke	2.789	0.000
Congestive heart failure	3.622	0.000
Renal failure	2.439	0.000
Previous ICH	2.115	0.197
Previous TIA	1.752	0.068
Previous PCI	1.850	0.000
Previous CABG	3.446	0.000
Peripheral artery disease	2.265	0.001
PCI during index hospitalization	0.358	0.000
CABG during index hospitalization	0.481	0.003
COPD	1.535	0.040
Angina pectoris	1.935	0.000
Heart failure symptoms during index hospitalization	1.397	0.011
Ischemic stroke during index hospitalization	5.921	0.002
Recurrent MI during index hospitalization	0.413	0.212
Aspirin treatment at discharge	0.907	0.638
OAC treatment at discharge	1.128	0.349
Betablocker treatment at discharge	0.778	0.111
Diuretics treatment at discharge	2.471	0.000
ACE inhibitor treatment at discharge	0.757	0.012
AT2-receptor antagonist treatment at discharge	1.237	0.123
Lipidlowering therapy at discharge	0.363	0.000
Clopidogrel treatment at discharge	1.467	0.001
Ticagrelor treatment at discharge	0.522	0.000
Prasugrel treatment at discharge	0.337	0.001
P2Y12-inhibitor treatment at discharge	0.815	0.114
Non-insulin dependent DM at discharge	0.727	0.213
Insulin dependent DM at discharge	2.703	0.000

*NSTEMI* non ST-segment elevation myocardial infarction, *STEMI* ST-segment myocardial infarction, *CV* cardiovascular, *ICH* intracranial hemorrhage, *TIA* transitory ischemic attack, *CABG* coronary artery bypass graft, *PCI* percutanteous coronary intervention, *COPD* chronic obstructive pulmonary disease, *OAC* oral anticoagulation, *ACE* angiotensin converting enzyme AT2 angiotensin 2, *DM* diabetes mellitus.

**Table 7:** Risk factors associated with CV-death, MI and IS among 1379 patients discharged after hospitalization for ACS in a multivariable Cox regression analysis.

Variable	HR (95% CI)	p-value
Age	1.03 (1.02–1.04)	< 0.001
Diabetes mellitus	1.74 (1.36–2.22)	< 0.001
Angina pectoris	1.41 (1.08–1.85)	0.012
CHF	1.56 (1.11–2.21)	0.011
Prior IS	1.46 (1.03–2.07)	< 0.001
Prior PCI	1.41 (1.00–2.00)	0.049
Prior CABG	1.82 (1.34–2.49)	< 0.001
PCI during index hospitalization	0.58 (0.44–0.77)	< 0.001
CABG during index hospitalization	0.51 (0.31–0.85)	0.010
Diuretics treatment at discharge	1.32 (1.04–1.68)	0.024
Lipid lowering therapy at discharge	0.69 (0.51–0.94)	0.017

*CHF* congestive heart failure, *IS* ischemic stroke, *PCI* percutaneous coronary intervention, *CABG* coronary artery bypass graft.

Competing risk analysis according to Fine and Gray did not alter the result of the multivariable analysis.

## Discussion

This was a retrospective observational study in a contemporary, population-based and unselected cohort of ACS-patients, with a long follow-up period, reporting long-term cardiovascular outcomes after ACS. To our knowledge, there are no comparable studies reporting outcomes in absolute numbers. Almost one out of three patients died for cardiovascular reasons or had a recurrent ischemic event during the study period. Over 40% of all events and more than half of all MI’s occurred during the first year. IS events were more evenly distributed, with nearly 1/3 occurring during the first year.

Compared with the Cardiovascular risk in post-myocardial infarction patients: nationwide real-world data demonstrate the importance of a long-term perspective study^6^, which was a Swedish nationwide register based study (n = 97,254) to assess the incidence and risk factors of subsequent cardiovascular events in the Swedish post MI population, we found a lower event rate both short and long term. That study included AMI-patients surviving the first week after discharge, with a total follow-up of 4 years and a mean follow-up time of 2.5 years. The 1-year rate of the composite of cardiovascular (CV) death, recurrent MI and stroke was estimated to 18.3%, and the risk remained high in patients without an event during the first year, in the subsequent 2 years at about 20%, which is almost twice as high as in our material. The patient data upon which these results are based is comparable with ours in terms of a high median age and a relatively low frequency of revascularization during hospitalization. On the other hand, preexisting heart failure was clearly more prevalent (26.3%) compared with our cohort (5.3%) which might contribute to a higher event rate in general and CV mortality in particular. Moreover, in our study all end-points were adjudicated and we only included type-1 MI’s, which likely contributes to the observed lower event rate. Type-2 MI has been associated with a clearly higher mortality in previous reports and the proportion of type-2 MI in SWEDEHEART has been estimated to 7.1%^11^. Despite these possible explanations, the results differ substantially which highlights the need for hiqh-quality long-term follow-up studies to estimate CV risk in a contemporary, unselected ACS population.

In a large study comparing long-term outcomes after MI (3 years) 2002–2011 in nationwide registers from England, France, Sweden and USA in more than 100,000 patients > 65 years, the cumulative incidence of the composite of all-cause death, MI and stroke varied between 26% (France) and 36.2% (USA)^12^. These numbers are more at level with our results when taking into account that the end-point included all-cause death and that the studied populations had a high burden of comorbidities and a very high mean age of 77.5–78.6 years.

These results indicate that the risk is high during the first year, and remains high also in a longer time perspective, which highlights the importance of adequate secondary prevention and revascularization procedures.

Older age, diabetes mellitus, prior ischemic stroke, heart failure (treatment with diuretics at discharge), established coronary heart disease (prior PCI, CABG or angina) independently predicted increased risk for the primary endpoint in our material, whereas revascularization during index hospitalization and lipid-lowering therapy were associated with favorable outcome. The most important established risk factors of CV events are family history of CVD, smoking, physical inactivity, hypertension, hyperlipidemia, diabetes mellitus, and abdominal obesity.

In accordance with previous publications, older age conferred increased risk for recurrent cardiovascular events in this study^13^. An ageing population and increasing proportion of survivors of ACS should urge health care systems to direct their attention regarding secondary prevention not only to younger patients. Diabetes Mellitus has repeatedly been shown to forcefully increase the risk for cardiovascular disease^14^. It has also been linked to excess mortality after MI compared with patients without diabetes^15^, and ESC guidelines for non-ST-segment elevation acute coronary syndromes (NSTE-ACS) recommend that a multifactorial approach, with treatment targets should be considered in this subset of ACS-patients (grade of recommendation IIa)^16^. In our material, diabetes was the comorbidity with the strongest association of elevated long term ischemic risk after ACS, which in conjunction with previous evidence points at the importance of good metabolic control and intensive lipid lowering therapy^17^. There is vast evidence from clinical trials that lipid-lowering statin therapy reduces cardiovascular morbidity and mortality in both chronic and acute coronary syndromes^18,19^. In our study, lipid-lowering therapy was strongly associated with a reduced risk for recurrent events in the long term, which confirm the results from clinical trials and contribute to generalizability to a real-world ACS-population.

Ischemic stroke and heart failure have, as in this study, been identified previously as markers of elevated risk for recurrent ischemic events and death^6,13,20,21^.

Revascularization during index-hospitalization for ACS, both CABG and PCI, were strongly associated with better outcome in this study. Jernberg et al. reported that absence of revascularization was associated with increased cardiovascular risk^6^. An early invasive strategy, with coronary angiography followed by PCI if appropriate, has been shown to reduce cardiovascular events long-term after NSTE-ACS, compared with an initial conservative strategy^22,23^. In ST-segment myocardial infarction (STEMI), there is solid evidence that prompt revascularization reduces mortality and subsequent ischemic events^24,25^. Elderly patients are generally considered to have a greater burden of coronary disease and thereby more myocardial ischemia and seem to derive a relatively greater benefit from revascularization^26,27^. Given the high median age in this cohort, this seems to hold true for our patient population and has important implications for the often difficult clinical decision-making regarding revascularization in the care of elderly patients with ACS.

## Strengths and limitations

This unselected, single-center cohort, with the aim to study the long-term cardiovascular outcome after hospitalization for ACS, was relatively small, which is a limitation. 60.1% of patients were examined with echocardiography during hospitalization, which is a relatively low number and represents a limitation in terms of the multivariable analysis. However, the data collected can be considered to be of high quality as well as the adjudication of clinical endpoints. The outcome measures cardiovascular death, myocardial infarction and ischemic stroke are fairly standardized. We chose to only include type 1 MI’s, because we had the opportunity to evaluate every clinical event, and since a type 1 MI has a different prognostic impact than for example a type 2 MI. This might lead to lower event rates compared with register-based studies, in which it is hard to discriminate between infarction types. Due to the establishment of a PPCI-network in the region, patients received a different reperfusion treatment, with a clear dominance of PPCI from May 2015 until the end of follow-up, compared with a fibrinolysis dominated reperfusion treatment before May 2015, which might has affected outcomes.

## Conclusion

In an unselected cohort of ACS-patients, with a median follow-up time of 4.7 years, the risk for the composite of CV-death, MI and IS was 10.3% at 1 year and 28.6% during the entire study period.

Risk factors associated with an increased risk of the primary end-point were Diabetes Mellitus, prior revascularization, heart failure at discharge and prior IS. Associated protective factors were revascularization during index hospitalization and lipid lowering therapy at discharge.

## Data Availability

The datasets used and/or analysed during the current study available from the corresponding author on reasonable request.
